# Supplementary material for: The proximity of ideas: An analysis of patent text using machine learning
Source: PLoS One. 2020 Jul 9;15(7):e0234880. doi: 10.1371/journal.pone.0234880 (PMC7347140; doi:10.1371/journal.pone.0234880)
Supplement: S1 Appendix — (PDF) [file pone.0234880.s001.pdf]

S1 Appendix.

Text cleaning

Each abstract is stemmed to the root word (for example, "computer" to "comput"), and stop words (such as "and", "the") are removed. The first step in converting text to data is to represent words and documents in their simplest vector forms. For all algorithms besides Document Vectors, input into the algorithms involve the construction of a document-term matrix from all patents; each row is indexed by the document ID and each column represents a word in the vocabulary. A document row vector represents the count of the number of times the term appears in the document. For the terms, I drop all terms that appear in more than 10% of all patents, and those that appear in fewer than 20.[12] Of the resulting terms, I keep the most common 40,000, in order to maintain a manageable matrix dimensionality. Once all 2,306,041 patents have been transformed into a document-term matrix of dimension 2306041×40000, I proceed to transforming patents into a smaller dimensional vector representation using the methods described below. This procedure is commonly called the bag-of-words representation of text data.
