# Supplementary material for: The proximity of ideas: An analysis of patent text using machine learning
Source: PLoS One. 2020 Jul 9;15(7):e0234880. doi: 10.1371/journal.pone.0234880 (PMC7347140; doi:10.1371/journal.pone.0234880)
Supplement: S2 Appendix — (PDF) [file pone.0234880.s002.pdf]

S2 Appendix.

Document Vectors (Doc2Vec)

One recent advance in NLP which utilises neural networks is Document Vectors, introduced by [28]. This is a straightforward extension of the word2vec model of [26,27]. The word2vec model attempts to rectify one of the well-known problems of NLP: the inability of "one-hot" word vectors to account for word similarity. Typically, word vectors are represented as sparse vectors. For example, in a complete vocabulary of ["good", "fair", "fine"], the word good would be represented as the vector $[1, 0, 0]$, fair as $[0, 1, 0]$ and fine as $[0, 0, 1]$. Clearly, each of these vectors are orthogonal to each other and have a similarity of 0. Instead of using this class of word vectors, word2vec tries to represent words as dense vectors that encode such similarities; a word2vec vector for each of the three words ["good", "fair", "fine"] will have a high similarity.

The way that this is done is through looking at the context of a word. For example, for the sentence "Provides for unattended file transfers", the word "unattended" has the context ["Provides", "for", "file", "transfers"]. We want to represent each of these words as a vector of arbitrary dimension n. One way to account for context is to predict the context words given the target (Skip-gram); while another way is to predict the target word given the context (Continuous Bag-of-Words). Under Skip-gram, the optimization problem is to maximise the probability of any context word given the current center word. So the objective function is given by:

$$J(\theta) = -\frac{1}{V} \sum_{t=1}^{V} \sum_{-m \leq j \leq m} \log p(w_{t+j}|w_t)$$

Where θ represents all parameters: input vector ("one-hot") representation of each word, and the output word2vec representation of each word. m represents the length of the context window; for example $m = 1$ gives the context for "unattended" as ["for", "file"]. The objective function is minimized using stochastic gradient descent.

[12] Including very common and very infrequent terms may introduce noise and considerable increases in computation times.

Document Vectors, or Doc2Vec, extends word2vec merely by adding an additional variable, which will be treated as an additional context vector: document ID. For my data, this will be the patent number, which uniquely identifies every abstract document. Thus, including document ID as an additional word for each context generated from that document text will also generate a unique vector associated with the document, as well as the word vectors. Intuitively, the document vector will represent what was learned in other context windows belonging to the document text, outside of the present context window: that is, it "acts as a memory that remembers what is missing from the current context." ([28])

Such an approach has been shown to be extremely powerful in accurately capturing cross-word and cross-document similarity, which is why it is the main focus of my analysis. Other vector representations of patents that I use do not specifically optimize to capture such similarity using contexts.
