# Supplementary material for: The proximity of ideas: An analysis of patent text using machine learning
Source: PLoS One. 2020 Jul 9;15(7):e0234880. doi: 10.1371/journal.pone.0234880 (PMC7347140; doi:10.1371/journal.pone.0234880)
Supplement: S3 Appendix — (PDF) [file pone.0234880.s003.pdf]

S3 Appendix.

Latent Dirichlet Allocation Latent Dirichlet Allocation, first introduced by [25], is a method of Topic Modelling that assumes that a document can be represented as a linear distribution hidden variables called topics. It is a Hierarchical Bayesian hidden variables model. The Data Generating Process assumes that each topic is a linear distribution over terms in the corpus. For each document, which is a distribution over topics, each term is assumed to be generated by first drawing a topic, then drawing a term from that topic. Because this is an unsupervised method, the algorithm then jointly determines the topics distribution over terms and each document's distribution over topics. The LDA section below contains more details on the assumptions of the model. S1 Table shows a breakdown of selected topics' distribution over terms. **??** provides an example of the input and outputs of the algorithm.

The number of topics K is a parameter that is determined ex-ante; as per [48], the recommendation is that the model with the lowest log perplexity be selected, although there is not a universally agreed upon procedure. I fit a LDA model on a training subset of the same document-term matrix representing all patent abstracts with $20, 30, ..., 120$ topics. Then, the model was fit on the test set and the log-perplexity calculated. I selected $K = 60$ as it had the lowest log perplexity across the models.

A snippet from the resulting topics is shown in S1 Table, alongside the six highest probability terms in teach topic. The output I am interested in is the probability across each of the 60 topics of each patent document. I take this as the Topic Model vector representation of each patent.

Data generating process With probabilistic models, treat observations as outcomes of a data generating model and infer the hidden parameters of that model using posterior inference. Define a "topic" as a discrete distribution over a fixed vocabulary. Assume each topic is generated by drawing a distribution over terms in the vocabulary represented by the vector: $\boldsymbol{\beta_k} = (\beta_{k,1}, ..., \beta_{k,V}) \sim Dir(\eta)$. Additionally, asssume that each document d is generated by the following process:

1. Draw a vector distribution over topics: $\boldsymbol{\theta}_d = (\theta_{d,1}, ..., \theta_{d,K}) \sim Dir(\boldsymbol{\alpha})$
2. For each word $w_{d,n}$:
   (a) Draw a topic $k_{d,n} \sim Multinomial(\boldsymbol{\theta}_d)$
   (b) Draw a word based on that topic's distribution over the vocabulary $w_{d,n} \sim Multinomial(\boldsymbol{\beta}_{z_{d,n}})$

Then the posterior of the hidden variables, conditional on the observed words in each document, is given by:

$$p(\beta_{1:K}, \theta_{1:D}, z_{1:D} | w_{1:D}) = \frac{p(\beta_{1:K}, \theta_{1:D}, z_{1:D}, w_{1:D})}{p(w_{1:D})}$$

An inference algorithm is used to approximate the posterior. Thus, from the observed set of V vocabulary terms $w \in 1, ..., V$, the hidden topics $k \in 1, ..., K$ (a distribution over words in the vocabulary), and each document's distribution over topics $(\theta_{d,1}, ..., \theta_{d,K})$ are derived.
