# Supplementary figures and images for: The proximity of ideas: An analysis of patent text using machine learning
Source: PLoS One. 2020 Jul 9;15(7):e0234880. doi: 10.1371/journal.pone.0234880 (PMC7347140; doi:10.1371/journal.pone.0234880)

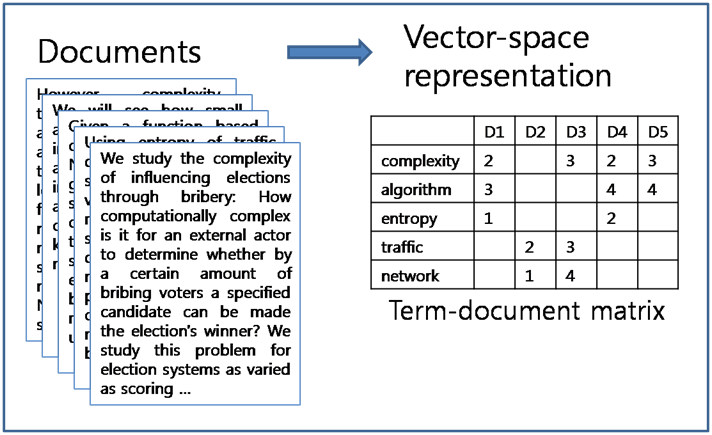

Supplement: S1 Fig — (TIFF) [file pone.0234880.s004.tiff]

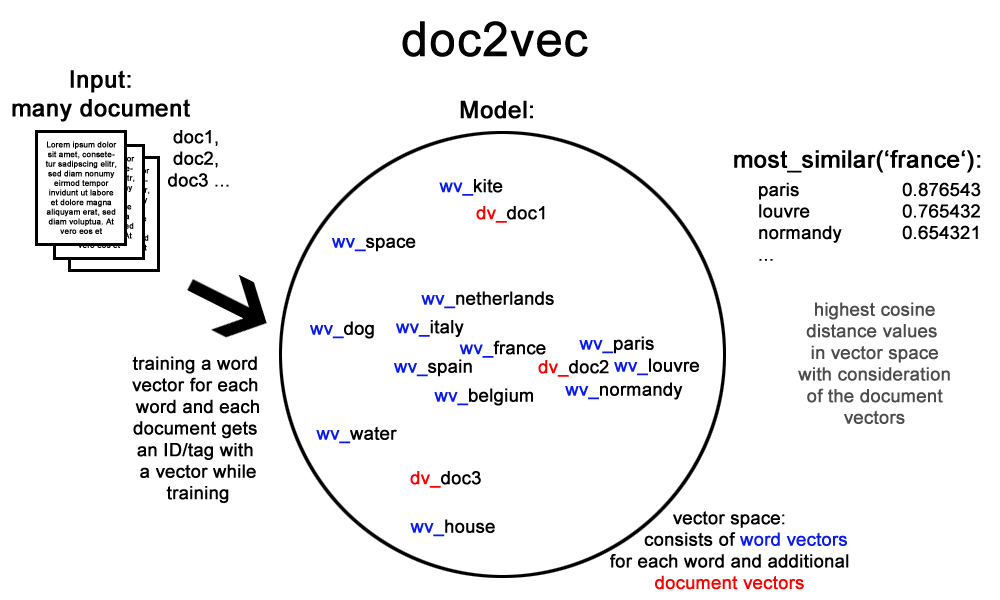

Supplement: S2 Fig — (TIFF) [file pone.0234880.s005.tiff]

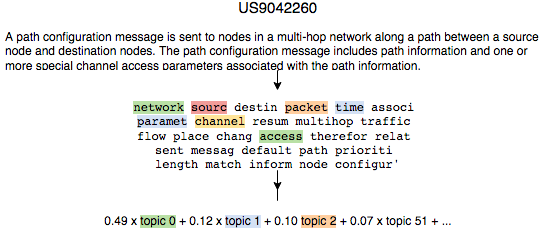

Supplement: S3 Fig — (TIFF) [file pone.0234880.s006.tiff]
