# Supplementary material for: The proximity of ideas: An analysis of patent text using machine learning
Source: PLoS One. 2020 Jul 9;15(7):e0234880. doi: 10.1371/journal.pone.0234880 (PMC7347140; doi:10.1371/journal.pone.0234880)
Supplement: S1 Table — (PDF) [file pone.0234880.s007.pdf]

S1 Table. Selected Topics as outputted by LDA. Description added post hoc.

Topic	Distribution over terms	Description
0	0.040*network + 0.039*inform + 0.033*comput + 0.031*communic + 0.028*user + 0.027*memori	Networks & Coding
2	0.066*time + 0.057*sensor + 0.040*detect + 0.032*event + 0.031*paramet + 0.027*level	Monitoring & Coding
11	0.116*power + 0.068*voltag + 0.049*output + 0.045*circuit + 0.026*suppli + 0.026*transistor	Electronics
36	'0.071*composit + 0.059*polym + 0.049*weight + 0.041*coat + 0.018*resin + 0.016*c	Polymers, Chemicals
53	'0.065*metal + 0.065*solut + 0.037*ion + 0.036*carbon + 0.032*concentr + 0.023*reaction	Metals, Chemicals
