# Supplementary material for: The proximity of ideas: An analysis of patent text using machine learning
Source: PLoS One. 2020 Jul 9;15(7):e0234880. doi: 10.1371/journal.pone.0234880 (PMC7347140; doi:10.1371/journal.pone.0234880)
Supplement: S2 Table — (PDF) [file pone.0234880.s008.pdf]

S2 Table. Regression results for JTH replication under different control selection methods. Localization estimates ($\hat{\beta}_1$) are the row values with standard errors in parentheses beneath. Sample sizes vary due to the inability to find control patents under certain methods of selection. Controls in the regression model include year and primary class fixed effects.

	1975-85	1985-95	1995-05
Control Selection: Standard JTH	0.2422***	0.2849***	0.2968***
	(0.0074)	(0.0051)	(0.0041)
N	58647	107358	185154
Adjusted R^2	0.03	0.05	0.05
Control Selection: Similarity	0.2029***	0.2681***	0.2621***
	(0.0100)	(0.0067)	(0.0054)
N	36917	67332	117137
Adjusted R^2	0.02	0.03	0.04
Control Selection: Lawyer	0.0806***	0.0935***	0.1150***
	(0.0136)	(0.0086)	(0.0069)
N	22914	51837	85855
Adjusted R^2	0.02	0.03	0.03
Controls: Year and Primary Class FE			
