# Supplementary material for: The proximity of ideas: An analysis of patent text using machine learning
Source: PLoS One. 2020 Jul 9;15(7):e0234880. doi: 10.1371/journal.pone.0234880 (PMC7347140; doi:10.1371/journal.pone.0234880)
Supplement: S4 Table — (PDF) [file pone.0234880.s010.pdf]

S4 Table. Regression estimates for the localization of idea proximity of patent claims text. Represents how many S.D.s more similar patents within cluster (location-technology field) are compared to across clusters. Standard errors of estimates are reported in parentheses below. A separate sample is computed for each definition of technology field at the industry and primary class level.

	1975-85	1985-95	1995-05	2005-15
Technology Field: NAICS	0.0406***	0.0488***	0.0523***	0.0489***
	(0.0051)	(0.0042)	(0.0034)	(0.0031)
N	194092	282099	443884	578054
Adjusted R^2	0.08	0.06	0.06	0.08
Technology Field: Primary Class	0.0393***	0.0402***	0.0468***	0.0425***
	(0.0062)	(0.0049)	(0.0038)	(0.0033)
N	171859	252881	407173	537875
Adjusted R^2	0.10	0.09	0.08	0.13
Controls: Year, PC, MSA, Examiner, Lawyer Match and FEs				
