# Supplementary material for: The proximity of ideas: An analysis of patent text using machine learning
Source: PLoS One. 2020 Jul 9;15(7):e0234880. doi: 10.1371/journal.pone.0234880 (PMC7347140; doi:10.1371/journal.pone.0234880)
Supplement: S5 Table — (PDF) [file pone.0234880.s011.pdf]

S5 Table. Comparison of similarity and backward citation overlap for new patents using new terms.

Target's Citations	Sim DocVecs First Year	Num Common Cited First Year, Num Pats	Pct Common Cited, First Year, Num Pairs			
gui	0.09	0.00	0.00	1992	1348	796727
lun	0.12	0.00	0.00	1995	415	63044
asic	0.11	0.00	0.00	1987	198	16716
url	0.10	0.01	0.00	1995	111	4847
serd	0.12	0.02	0.00	1998	75	1929
chat	0.10	0.00	0.00	1992	9	1299
bist	0.12	0.00	0.00	1990	42	810
femto	0.15	0.04	0.00	2007	27	563
angst	0.16	0.00	0.00	1994	40	549
mcm	0.10	0.00	0.00	1991	32	440
www	0.15	0.01	0.01	1995	9	291
efus	0.12	0.02	0.00	2000	22	201
femtocel	0.22	0.09	0.00	2007	8	198
adenovir	0.26	0.03	0.00	1993	15	98
cyclin	0.18	0.00	0.00	1991	13	80
n 1	0.13	0.00	0.00	1998	13	63
dvd	0.16	0.00	0.00	1996	10	44
websit	0.18	0.00	0.00	1996	10	42
gpu	0.05	0.00	0.00	2001	9	36
pcie	0.21	0.00	0.00	2004	9	35
